# Investigating the relationship between socioeconomic status and domestic violence against women in Isfahan, Iran in 2021: A cross‐sectional study

**DOI:** 10.1002/hsr2.1277

**Published:** 2023-05-18

**Authors:** Niloofar Dabaghi, Mostafa Amini‐Rarani, Mehdi Nosratabadi

**Affiliations:** ^1^ Department of Health and social welfare, School of Management and Medical Information Sciences Isfahan University of Medical Sciences Isfahan Iran; ^2^ Health Management and Economics Research Center Isfahan University of Medical Sciences Isfahan Iran; ^3^ Social Determinants of Health Research Center Isfahan University of Medical Sciences Isfahan Iran

**Keywords:** Iran, socioeconomic status, violence against women

## Abstract

**Background and Aims:**

Domestic violence can include controlling or coercive behaviors and acts, as well as physical, sexual, psychological, and financial elements. Given the significance of domestic violence against women and its complications, this study looked into the relationship between socioeconomic status and domestic violence against women in Isfahan in 2019.

**Methods:**

In 2021, a cross‐sectional study of 427 married women referred to comprehensive health centers in Isfahan, Iran, was carried out. The available sampling method was chosen. To collect data, a domestic violence questionnaire and a socioeconomic status index were used. The data were analyzed using SPSS and Latent GOLD software.

**Results:**

The average age of the women in this study was 33.21, 37% worked, and 63 were housewives. Based on Latent class analysis method, women were classified into two groups of high or low socioeconomic status class. The findings revealed a significant relationship between socioeconomic status and different types of violence against women, including light physical violence, emotional violence, verbal violence, and sexual violence (*p* < 0.05).

**Conclusion:**

The findings revealed that there is a significant relationship between socioeconomic status and domestic violence against women in Isfahan, with women from lower socioeconomic backgrounds being more vulnerable to violence. Given the prevalence of violence against women in the family and its consequences, policy makers should look for the causes of this type of violence as well as solutions to reduce this health and social problem. Factors such as the expansion of counseling and treatment centers in health care facilities, as well as education and life skills training, are particularly important in reducing this phenomenon in society.

## 1 INTRODUCTION

Domestic violence is a social issue that has occupied the minds of social researchers for a long time and is not only affected by different aspects of human life, but will also affect them.^1^

Domestic violence, which can occur in relationships or between partners, is frequently used as a term for intimate partner violence, which is committed by one of the people in an intimate relationship against the other.

Violence against women refers to acts of aggression performed predominantly or only by men or boys against women or girls. Such violence, which is frequently categorized as a type of hate crime.^2^ and is frequently directed toward women or girls solely for being female, can take many different forms.

Domestic violence is a phenomenon in which a woman is subjected to violence and violation of her rights by the other sex because of her gender. If this type of behavior occurs within the framework of the family and between the husband and wife, it is considered to be domestic violence.^3^ Violence against women is a public health and social issue with numerous physical and psychological consequences. According to the findings of various studies, all types of pains, digestive problems, bleeding, abortion, and damage to body organs such as eardrum rupture, blindness, and broken limbs are common among victims of violence.^4^, ^5^ This problem also causes depression, anxiety, posttraumatic stress disorder, irritable bowel syndrome, headache, suicidal thoughts, panic attacks, and other symptoms.^6^, ^7^

In comparison to other regions like America (30%) and Europe (25%), South East Asia (38%), and Africa (37%) were shown to have greater regional prevalence rates of intimate partner violence.^8^ According to the Iranian Statistics Center, the total number of cases of physical examination of spouse abuse claimants in 2019 indicates that during this year, 80,187 cases of physical examination were conducted by the forensic doctor, with the majority of claimants being women. There were 77,000 cases of physical spousal abuse investigated in these examinations, with 2900 men claiming physical spousal abuse.^9^

In fact, women claiming physical spousal abuse accounted for 96% of all forensic medical examinations related to physical spousal abuse claimants in 2013, while men claiming physical wife's abuse accounted for 4%.^10^, ^11^

Although domestic violence against women exists in all societies and socioeconomic classes, it appears that women in lower economic and social situations face more violence, and factors such as low literacy, low income, poverty, a lack of resources, and problems caused by having children are significant in people who engage in or are victims of violence.^12^ The nature of this phenomenon varies by country; for example, in Ghana, violence against women can have religious, social, cultural, sexual, physical, emotional, psychological, or economic aspects.^13^

In reviewing the research done in Iran on this subject, various factors have been found to be effective in relation to domestic violence and in reducing or increasing it, such as increasing age,^14^ higher education for women,^15^ and female employment.^16^ In there has been an increase in violence in some cases, a decrease in violence in others, and no connection with violence in some cases.^17^

As can be seen from the studies, the majority of these studies have investigated the various dimensions of violence and the consequences associated with it, while an aspect related to the socioeconomic index has not been investigated.^18^ The socioeconomic status of the household is regarded as one of the most important variables influencing domestic violence.^19^ This variable influences the mental and social health of community members throughout their lives, and having a suitable socioeconomic status through mechanisms Biological, psychological, and social factors can influence a person's decision to adopt healthy behaviors, form appropriate social and family relationships, and experience lower rates of social harm, including domestic violence against women.^20^, ^21^

The prevalence of domestic violence and specifically violence against women in the provinces of Iran is different and even in small geographical areas. Since the metropolis of Isfahan has a distinct cultural, social, and economic context, it includes various ethnic groups and social groups, including families with different socioeconomic statuses and immigrants, it was deemed necessary to investigate this phenomenon. As far as we know, no study on the relationship between the index of socioeconomic status and domestic violence has been conducted in Iran or in Isfahan. As a result, the purpose of this study was to investigate the relationship between socioeconomic status and the experience of various types of violence among women in Isfahan.

## 2 METHOD

### 2.1 Setting and sampling

This study was conducted in 2019 on married women living in Isfahan City. The studied population was 427 married women referring to comprehensive health centers. To better understand the questionnaire, only married women who were literate were included in the study. To obtain the research samples, after approving the project and obtaining the necessary permits, the researcher went to the desired centers and selected married women who met the conditions for entering the study, and after explaining the research objectives and Emphasizing the confidentiality of information and obtaining their consent, invited them to participate in the study and complete the questionnaire.

Sampling was carried out in such a way that the city of Isfahan was divided into five parts: north, south, center, east, and west (This classification was done according to the cultural, economic, and social context of Isfahan as well as the division of Isfahan municipality.) then, three comprehensive health centers were chosen from each region. It was chosen at random, and approximately 30 questionnaires were completed in each center. Due to the presence of COVID‐19 and vaccination in a number of centers, the researcher was only allowed to visit the centers recommended by the experts. Based on this, we used available sampling in this study at the center selection stage. But after that, women were included in the study through random classification sampling. In the end, 15 centers (3 centers from each geographical region of Isfahan) were randomly selected.

We included all Isfahan women seeking care at comprehensive health centers in our sample. In fact, people go to comprehensive health centers to receive normal healthcare services even though they may not necessarily have a specific disease and have health records.

### 2.2 Inclusion and exclusion criteria

Women who were married, at least 1 year had passed since their marriage, were between the ages of 18 and 49, and were not going through a divorce were all required to meet the inclusion criteria. Women who were divorced or whose spouses had passed away at the time of the survey were excluded.

### 2.3 Measurement

To collect information, a checklist related to demographic variables, *questionnaire* of *violence against women* and socioeconomic status index were used.

Checklist related to demographic variables includes information such as woman's age, husband's age, and woman's age at the time of marriage, woman's employment status, husband's employment status, woman's education level, and husband's education level.

### 2.4 Violence against women questionnaire

The questionnaire of violence against women was designed by Alipour et al.^22^ and includes 19 questions that cover five dimensions of severe physical violence, light physical violence, emotional violence, verbal violence, and sexual violence. Each of the components of violence against women questionnaire was scored as never (1), very little (2), little (3), moderate (4), high (5), and very high (6). The score of each dimension was obtained from the sum of its items, and the total score of the questionnaire was obtained by summing the five dimensions.

Validity and reliability of the questionnaire have been investigated by Alipour et al. exploratory factor analysis was used to determine validity and Cronbach's *α* coefficient was used to determine reliability. The value of Cronbach's *α* coefficient for this questionnaire was 0.86.^22^

### 2.5 Construction of socioeconomic status

Latent class analysis method^23^ was used to construct the socioeconomic status index of the household. In this study, property indicators and social variables were used to construct SES as follows:

Owning a television, owning a car, owning a dishwasher, owning a computer/laptop, owning a microwave, owning a smart cell phone, owning a home, education of a woman, education of a husband.

The variables of wife's and husband's education were measured at rank level (primary education, middle school, high school, and university education including diploma and higher). Other variables were measured at nominal level (have/does not have).

Based on this, four models were estimated and finally, the model with two hidden classes was selected based on the relatively low values of the Bayesian Information Criterion (BIC) and Cluster error, as well as the corresponding graphs. The studied women were classified into two groups of high/low socioeconomic class.

### 2.6 Data analysis

*T*‐test was used to compare the means of dimensions of violence against women (light physical violence, severe physical violence, verbal violence, emotional violence, and sexual violence) between the high and lower socioeconomic classes of the household, in addition to describing the qualitative variables in the form of number and percentage. The statistical significance level was set at 0.05. SPSS (SPSS Inc., version 22.0) and Latent GOLD (Statistical Innovations Inc., Version 5.1) software were used to analyze the data.

### 2.7 Findings

#### 2.7.1 Descriptive statistics and sample characteristics

The results of the description of the research data indicate that the average age of women was 33.29 and the average age of their husbands was 36.9826. The average age of women at the time of marriage was 31.20. 37% of women were working and 63 were housewives. Regarding the level of education, the highest percentage was related to the educational level of diploma with a frequency of 34% (Table 1).

**Table 1:** Socio‐demographic characteristic of the study population.

		Wifes		Husband	
Characteristic of participants		Number (Mean)	Percentage (SD)	Number (Mean)	Percentage (SD)
Age	At the time of marriage	25	54	‐	‐
	At the time of sampling	32.2	8.9	36.9	9.3
	Employed	158	37.0	‐	‐
	Housewife/Unemployed	269	63.0	78	18/3
Emolument status	Seasonal worker	‐	‐	33	7.7
	Industrial worker	‐	‐	189	44.3
	Employee	‐	‐	12	2.8
	The employer	‐	‐	115	26.9
	Elementary	9	2.1	17	4.0
	Middle school	34	8.0	59	13.
	Diploma	145	34.0	133	31/1
Educational status	Associate degree	105	24.6	82	19.2
	Bachelor's degree	94	22/0	107	25.1
	Master's degree	27	6/3	23	5.4
	Doctoral degree	13	3/0	6	1.4

**Figure 1:**
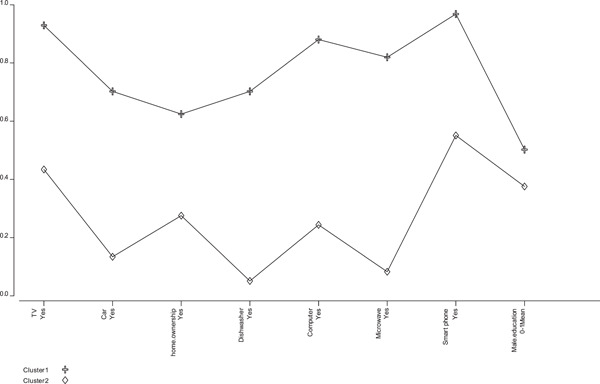
Two‐cluster model of socioeconomic status index.

In the part of analytical findings, the index of the socioeconomic status of the household was created using the latent class analysis method, based on four models which were estimated, finally the model with two hidden classes were selected according to the relatively low values of the BIC and Cluster error and related graphs. Therefore, the studied women were classified into two groups of high socioeconomic class and low socioeconomic class (Figure 1).

In the next step, the *t*‐test of two independent samples was used to compare the relationship between the types of violence against women based of SES status of Household. The results showed that there is a relationship between socioeconomic status and light physical violence, emotional violence, sexual violence, and verbal violence (*p* < 0.05), but no relationship was observed between socioeconomic status and severe physical violence (Table 2).

**Table 2:** Comparison of the means of types if violence against women based on high/low socio‐economic status (SES).

Variable	Groups	Number	Mean	Standard deviation	Freedom degree (*df*)	Amount of *T*	Significant level (*p*‐value)
Severe physical violence	High SES	254	5.08	1.22	425	1.45	0.14
	Low SES	173	25.26	1.36			
Light physical violence	High SES	254	8.57	2.17	425	3.10	*p* < 0.001
	Low SES	173	9.22	2.15			
Emotional violence	High SES	254	10.45	2.81	425	4.62	*p* < 0.001
	Low SES	173	11.69	2.58			
Verbal violence	High SES	254	849	2.62	425	4.72	*p* < 0.001
	Low SES	173	9.76	2.35			
Sexual violence	High SES	254	7.14	2.19	425	4.81	*p* < 0.001
	Low SES	173	8.15	2.07			

## 3 DISCUSSION

Based on the results obtained from this study, there is generally a relationship between socioeconomic status and domestic violence against women in Isfahan, Iran. Social control theories, the frustration‐aggression theory, resource theory, and feminist theory were used to interpret research hypotheses.

According to the *T*‐test, the first hypothesis, “a significant relationship between the socioeconomic status of the household and severe physical violence against women,” was not statistically confirmed. It was explained using social control theory. This theory emphasizes the existence of human crime and violent behavior. In fact, people frequently use force and power to achieve their objectives or gain authority over others. Because people tend to commit crimes and engage in abnormal behavior without the presence of social constraints, society must devise a mechanism to monitor them. Violence occurs in the family as a result of the absence of government regulatory institutions.^24^

According to social control theory, when formal or informal social controls on violence are reduced, the rate of violence increases, so children grow up in families where parents disagree or have harsh educational and disciplinary methods. They are also more likely to commit violent acts in the future. In addition, there are no official monitoring institutions in the private area of the house, which facilitates the occurrence of violence in the family.

Our finding was consistent with Ince‐Yenilmez’ study.^25^ In fact, there may be other factors related to the socioeconomic status of the family, such as the presence of women in the labor market, the educational status of women, changing cultures and attitudes toward sever physical violence against women in the society, which did not make this relationship meaningful.

The second hypothesis, “a significant relationship between the socioeconomic status of the household and light physical violence against women,” was confirmed by *T*‐test. According to Homans’ frustration‐aggression theory,^26^ when a person is frustrated, he is likely to exhibit aggressive behavior. If the main source of frustration cannot be addressed for whatever reason, another goal can take its place. It appears that the pressures and economic problems, as well as the failure to meet the basic needs of the family, the lack of fair distribution of wealth and the possibility of access to sources of wealth creation, employment, and education, as well as the difficulty and load of personal responsibilities, cause men to rebel against women's wishes. This condition can cause individual to lose patience, become angry, anxious, moody, depressed, and finally violent.^27^, ^28^

Women, according to William Good's theory of resources,^29^ are economically more dependent on their husbands. That is, they have fewer options and resources for dealing with their violent spouse's behavior. Couples with equal power in the family, on the other hand, have less conflict. The decision‐making method^30^ was used in this theory to determine the authority of a person with resources in the family. One of the family members is usually asked who makes the final decision or has the last word in the family when using this method. Specific questions about family decisions are posed. As a result, the person who has the ultimate word has more authority and power. Anyone in the family who has more access to the family's resources can force other members to act in the direction of their desires; as a result, women of low social and economic class who are economically dependent on their husbands and also have fewer resources to deal with the violence of their wives are more likely to be abused. Such findings are consistent with the findings of Bazazbanisi et al.^31^ and Ler et al.^32^

The association between lower socioeconomic status and higher prevalence of domestic violence against women can be explained by lack of access to resources and increased acceptance of violence. Consistent with our study, Semahegn and Mengistie^33^ and Alhabib et al.^34^ concluded that a history of abuse reinforces the normative nature of violence, thus making men more likely to perpetrate and women more likely to accept violence.

However, our findings were contrary to the findings of Pambè et al.^35^ and Vyas et al.^36^ In these two studies, factors such as women's participation in decision‐making, community‐level influences, the existence of value beliefs, financial independence, and high human capital in women were able to make the relationship between economic‐social status and violence against women not to be significant.

The third hypothesis, “a significant relationship between household socioeconomic status and emotional violence against women,” was confirmed by the *T*‐test. It should be noted that the scope of violence encompasses a wide range of human behavior. In Iran, Physical violence has decreased as society's culture has changed, but emotional and psychological violence has increased.^37^ Educational, legal, and supportive strategies such as empowering families, among others, have been reported to improve skills in dealing with spousal violence. Our findings are consistent with studies of Afkhamzadeh et al.^38^ and Asadi et al.^14^ Beside, in two studies conducted in Malaysia, Othman et al.^39^ and Haron et al.^40^ reported that emotional abuse was as the most common form of violence against women in Malaysia.

In a national household survey study of 36 countries in developing countries, WILSON.^41^ shows a negative association between household socioeconomic status and the annual incidence of emotional violence against women.

*T*‐test confirmed the fourth hypothesis, “a significant relationship between the socioeconomic status of the household and verbal violence against women,” to explain the results. It can be said that in traditional societies, people and women who are victims of violence, particularly low‐income women, have a special opinion about men's violent behavior and consider their living conditions to be normal. In this situation, men's disgusting behavior is justified. Among them, are women who should keep their husband's shadow over their children and themselves by tolerating men's behavior in family life and not divorcing. As a result, patriarchal beliefs, violence against women is considered natural in society, and according to feminist theory, the main reason for violence is the existence of patriarchal structures in society, and the social and economic structure of societies is based on belittling, insulting, and humiliating women.^42^ Our findings are consistent with the findings of Daruwalla et al.^43^ and Ler et al.^32^

The fifth hypothesis, “a significant relationship between household socioeconomic status and sexual violence against women,” was confirmed by *T*‐test. It should be noted that the culture of violence in society is an important factor to consider when explaining violence, particularly sexual violence. In our culture, discussing sexual issues is sometimes frowned upon. Because of the emphasis on veiling and modesty, many women have been forced to avoid expressing their desires and desired behavior, even in the most private setting with their husbands. This, along with many other taboos in women's minds, can lead to dissatisfaction with sex and, as a result, sexual violence against women. The results of this hypothesis in our study are in line with the research results of Grose et al.^44^ and Moazen et al.^45^ Beside, in consistent with our study, Bhona et al.^46^ found that need for work and survival can often expose women to situations of vulnerability and disrespect by the intimate partners resulting in sexual violence.

In the resource theory, VanderEnde et al.^47^ also state that men in households with low SES rely more heavily on violence as a means of controlling their partners in domestic decision‐making than do they have other resources (such as wealth or educational qualifications).

Overall, the results of our study are consistent with previous WHO^48^ report showing higher prevalence of domestic violence in low‐ to middle‐income countries compared to wealthier countries. The economic status of household may have influenced women's empowerment, increased literacy, women's economic dependence, and more gender‐equal norms in society.

### 3.1 Limitations

In this study, we've faced some limitations. The main limitation of our work was women's reluctance to fill out the questionnaire due to the sensitivity of the questions about sexual violence. According to this case, the researcher assured the women that the questions would be kept confidential and that the research results would only be used for policy and legislation to improve women's psycho‐social health.

## 4 CONCLUSION

Based on the findings of our study and a review of the empirical literature on the prevalence of violence against women, it can be concluded that domestic violence is not a new phenomenon that has emerged in the modern era, but has existed for a long time in various social and economic classes with varying types and levels. According to the findings of this study, women in lower socioeconomic positions are more vulnerable to violence, which may be attributed to psychological pressures caused by economic problems in people's lives, which, of course, necessitates additional research in this area. As a result, the findings indicate that the phenomenon of violence against women as a social problem in various strata of women's society requires extensive research, and given the high number of violence against women in the family and its impact on life, it is necessary to look for the roots of this type of violence and find solutions to reduce this health and social problem. It appears that to prevent and solve this problem, women's attitudes toward violence against women should be changed first, followed by an increase in women's selfefficacy and empowerment.

Furthermore, it can be stated that factors such as teaching life skills and promoting a culture of counseling among couples, among others, are especially important in reducing this phenomenon in society; additionally, with the expansion of counseling and treatment centers in health centers and women's awareness, it may be possible to reduce the incidence of domestic violence and the physical and mental complications caused by it.

## AUTHOR CONTRIBUTIONS

**Niloofar Dabaghi**: Conceptualization; formal analysis; writing—review and editing. **Mostafa Amini‐Rarani**: Methodology; writing—review and editing. **Mehdi Nosratabadi**: Conceptualization; methodology; writing—review and editing.

## CONFLICT OF INTEREST STATEMENT

The authors declare no conflict of interest.

## ETHICS STATEMENT

The study received the required ethics approval from the Isfahan University of Medical Sciences Research Ethics Committee, Isfahan Iran, with Ethics Code No. IR.MUI.REC.1399.388. Also, participants sign a written informed consent in which they have been assured that their identities and responses will be anonymous and that participants’ data will be kept confidential as possible. All authors have read and approved the final version of the manuscript. The corresponding author has full access to all of the data in this study and takes complete responsibility for the integrity of the data and the accuracy of the data analysis.

## TRANSPARENCY STATEMENT

The lead author Mehdi Nosratabadi affirms that this manuscript is an honest, accurate, and transparent account of the study being reported; that no important aspects of the study have been omitted; and that any discrepancies from the study as planned (and, if relevant, registered) have been explained.

## Data Availability

Data are available on reasonable request. The data that support the findings of this study are available from the corresponding author. However, they are not publicly available due to privacy and ethical restrictions.
